# RNase I_f_ -treated quantitative PCR for dsRNA quantitation of RNAi trait in genetically modified crops

**DOI:** 10.1186/s12896-018-0413-6

**Published:** 2018-01-17

**Authors:** Po-Hao Wang, Greg Schulenberg, Shannon Whitlock, Andrew Worden, Ning Zhou, Stephen Novak, Wei Chen

**Affiliations:** Dow AgroSciences LLC, 9330 Zionsville Rd, Indianapolis, IN 46268 USA

**Keywords:** RNAi, GM, RNase I_f_ –qPCR, Western corn rootworm (WCR)

## Abstract

**Background:**

RNA interference (RNAi) technology has been widely used to knockdown target genes via post-transcriptional silencing. In plants, RNAi is used as an effective tool with diverse applications being developed such as resistance against insects, fungi, viruses, and metabolism manipulation. To develop genetically modified (GM) RNAi traits for insect control, a transgene is created and composed of an inversely-repeated sequence of the target gene with a spacer region inserted between the repeats. The transgene design is subject to form a self-complementary hairpin RNA (hpRNA) and the active molecules are > 60 bp doubled-stranded RNA (dsRNA) derived from the hpRNA. However, in some cases, an undesirable intermediate such as single-stranded RNA (ssRNA) may be formed, which is not an active molecule. The aforementioned characteristics of RNAi traits lead to increase the challenges for RNAi-derived dsRNA quantitation.

**Results:**

To quantify the dsRNA and distinguish it from the ssRNA in transgenic maize, an analytical tool is required to be able to effectively quantify dsRNA which contains a strong secondary structure. Herein, we develop a modified qRT-PCR method (abbreviated as RNase I_f_ -qPCR) coupled with a ssRNA preferred endonuclease (i.e., RNase I_f_). This method enables the precise measurement of the active molecules (i.e., dsRNA) derived from RNAi traits of GM crops and separately quantifies the dsRNA from ssRNA. Notably, we also demonstrate that the RNase I_f_ -qPCR is comparable to a hybridization-based method (Quantigene Plex 2.0).

**Conclusions:**

To our best knowledge, this is the first report of a method combining RNase I_f_ with modified qRT-PCR protocol. The method represents a reliable analytical tool to quantify dsRNA for GM RNAi crops. It provides a cost-effective and feasible analytical tool for general molecular laboratory without using additional equipment for other methods. The RNase I_f_ -qPCR method demonstrates high sensitivity (to 0.001 pg/ μL of dsRNA), precision and accuracy. In this report, we demonstrated the deployment of this method to characterize the RNAi events carrying v-ATPase C in maize during trait development process. The method can be utilized in any application which requires the dsRNA quantification such as double-stranded RNA virus or sprayable dsRNA as herbicide.

**Electronic supplementary material:**

The online version of this article (10.1186/s12896-018-0413-6) contains supplementary material, which is available to authorized users.

## Background

RNA interference (RNAi) mediates a posttranscriptional gene-silencing (PTGS) mechanism via the process of exogenous double-stranded RNA (dsRNA) silencing complementary to endogenous mRNA in the cell [1]. Due to its high specificity in knockdown gene expression, RNAi has been developed into a widely used tool in plants for a variety of applications such as nematode resistance [2], insect resistance [3], fungi resistance [4], viral resistance [5], or parasitic weed resistance [6]. The use of RNAi as a pest control tool was first discovered and tested using the non-parasitic nematode *Caenorhabditis elegans* [7] and has been extensively investigated in the past decades for the development of a new mode of action for genetic modified (GM) crops to control pests in addition to conventional *Bt* (*Bacillus thuringiensis*) traits (detailed reviews in [8, 9]).

The RNAi approach for insect control becomes a practical application in crop protection through the expression of hairpin RNAs (hpRNA) in GM crops to downregulate essential genes in target insects. Transgenic plants producing hpRNAs to control insect pests were first demonstrated in western corn rootworm (WCR) (*Diabrotica virgifera*) [3] and cotton bollworm (*Helicoverpa armigera*) [10]. Both studies used a transgenic RNAi approach to produce plant material for oral ingestion. This allowed the dsRNA to silence the essential genes of the targeted insects for effective insect control. The key attributes of a successful RNAi approach for insect control are: (1) Identification of essential genes and (2) sufficient amount of dsRNA for delivery to the insect.

In the study by Mao et al. (2007) plants engineered to express dsRNAs against the bollworm *CYP6AE14* gene showed effective larvae growth inhibition [10]. More interestingly, when the transgene was introduced into *Arabidopsis* dicer mutants (e.g. *dcl2*, *dcl3* and *dcl4*), it resulted in greater abundance of longer dsRNA, *CYP6AE14* gene in the insect is repressed more effectively by transgenic plant materials. This result suggests that a minimal length of dsRNA is required to achieve the optimal efficiency of repression of targeted genes in pests. Moreover, two recent studies demonstrated the length of dsRNA is critical to achieve the desired efficacy. In the study described by Bolognesi et al., different sequence lengths of dsRNAs against *Dv* Snf7 were tested in bioassays, a size cut-off of approximately 60 bp dsRNA is required to display efficacy against corn rootworm [11]. Another study reported by Li et al. also showed dsRNA against *Dv* v-ATPase C with at least 60 bp in length resulted in high levels of larval mortality [12]. In contrast, feeding the pooled 21-bp siRNAs that have homology to v-ATPase C do not cause any mortality in both WCR larvae and adults.

Interestingly, the gene encoding RNA-dependent RNA polymerase (RdRP), required for the secondary siRNA amplification and transitive RNAi effects, is absent in insects [13] but is present in nematodes and plants [14, 15]. The lack of the RdRP activity may implicate that any effect of RNAi is a direct result from oral ingestion of dsRNA. A recent study displays evidence that the RNAi effect in WCR is not attributed to the transitive RNAi. In contrast, the siRNA distribution is solely restricted to sequences within the initial target sequence regions [16]. Taken together, these results indicate that the sequence length and initial concentration of transgene-derived dsRNAs for oral ingestion are key attributes for insect control.

Since the length and concentration of dsRNA are critical attributes to achieve the efficacy for controlling WCR, a reliable analytical tool to quantify dsRNA is required for RNAi trait development. The approach of detecting dsRNAs can be challenging due to its strong secondary structure derived from self-complementary inverted repeats (IRs). The secondary structure of the RNA molecules is often unpredicted and is determined by specific base pairing interactions within the encoded nucleotide sequence [17]. In addition, due to the design of transgene cassette, transgene may form dsRNA variants; some RNA variants may lead to intermediate forms of ssRNA, leading to non-efficacious molecules.

Studies in viral biology have developed some methods to characterize viral dsRNA such as immunofluorescence analysis or conventional RT-PCR. Using the monoclonal dsRNA-specific mouse antibody, the dsRNA may be detected via immunoblot or immunohistochemistry [18, 19]. Moreover, studies in viral biology use high (e.g., 96 °C) and low temperature (e.g., 70 °C) to denature the dsRNA and ssRNA, respectively [20]. Recently, a hybridization-based method using the QuantiGene technology (Affymetrix) has been reported for effective dsRNA quantitation [21]. In this report, we developed a modified reverse transcription PCR (RT-PCR) by coupling the RNase I_f_ -treatment RNA prior to cDNA conversion (abbreviation: RNaseI_f_ -qPCR) which has proven to be both cost effective and feasible. RNase I_f_ is an endonuclease which has preferential activity to digest ssRNA over dsRNA. RNaseI_f_ -qPCR allows users to precisely distinguish dsRNA from ssRNA and effectively assay the dsRNA in a high-throughput manner. The reported method here may be easily adopted for any applications in the emerging field of RNAi-based insect pest control with the need for dsRNA quantitation.

## Methods

### Synthetic double-stranded RNA and single-stranded RNA of *Dv* v-ATPase C

In this study, we used RNAi carrying sequence targeting vacuolar H + -ATPase (V-ATPase) subunit C in western corn rootworm (WCR) (*Diabrotica virgifera*) for our testing. V-ATPase is a highly conserved enzyme complex that acidifies intracellular organelles through pumping protons across the plasma membranes and consists of catalytic V1 and membrane-bound V0 two subcomplexes [22]. V-ATPase C in WCR is one of the effective dsRNA target. Previous study has demonstrated the efficacy of dsRNA targeting *Dv* v-ATPase C [12]. To validate the method, a 149-bp sequence (nucleotide positions 9-157 of *Dv* v-ATPase C open reading frame (ORF)) was selected for dsRNA synthesis [12]. The dsRNA was synthesized (agroRNA, Seoul, Korea). For ssRNA, a 125-bp sequence overlapping with the 149-bp dsRNA region was selected for ssRNA synthesis. The ssRNA was synthesized through IDT DNA Technologies (Iowa). One hundred nanogram synthetic *Dv* v-ATPase C dsRNA was treated with DNase (Ambion, Life Technologies, Austin TX, USA) to eliminate DNA contamination. After inactivation of the DNase, the dsRNA was then diluted to 100 pg/μL. The synthetic ssRNA, without further DNase treatment, was diluted to 100 pg/μL with RNase free H_2_O. The 10-point standard dilution and mixtures of dsRNA, ssRNA and wild-type maize B104 leaf RNA were created at the different ratios as specified in Tables 1, 2 and 3.

**Table 1:** RNase I_f_ -qPCR assay RTL (Relative transcript level) and % recovery for 10-point dilution series of dsRNA and ssRNA

dsRNA							ssRNA					
Standard point	dsRNAamount (pg)	R9S RTL	R70RTL	70 RTL	MeasureddsRNAamount	% recovery	ssRNAamount (Pg}	R95 RTL	R70RTL	70 RTL	MeasuredssRNAamount	% recovery
I	6.67	138.64	0.03	2.44	8 08	121%	6.67	0.08	0.00	79.82	7.89	118%
2	3.33	S9.26	0.01	0.83	3.57	107%	3.33	0.06	0.00	32.71	3.32	100%
3	1.67	26.95	0.01	0.40	1.67	100%	1.67	0.00	0.00	15.67	1.62	97%
4	0.83	12.68	0.00	0.21	0.81	97%	0.83	0.00	0.00	7.03	0.75	89%
5	0.42	4.97	0.00	0.08	0.33	19%	0.42	0.05	0.00	4.07	0.44	105%
6	0.21	2.28	0.00	0.05	0.16	75%	0.21	0./9	0.00	1./8	0.20	95%
/	0.10	1.44	0.00	0.03	0.10	96%	0.10	0.00	0.00	0.83	0.09	90%
8	o.os	0.81	0.00	0.01	006	110%	O.OS	0.10	0.00	0.43	0.05	95%
9	0.03	0.35	0.00	0.01	003	99%	0.03	0.06	0.00	0.23	0.03	105%
10	0.01	0.20	0.00	0.00	0.02	117%	0.01	0.05	0.00	0.12	0.01	110%

10-point standard synthetic dsRNAs and ssRNAs were prepared as the concentration shown. dsRNA/ssRNA amount (pg) is represented in a 10 μL qPCR reaction. The synthetic RNAs were analyzed using the RNase I_f_ -qPCR protocol described above. The relative transcript level (RTL) from different steps of RNase I_f_ -qPCR protocol is denoted as R95 RTL (95 °C RTL with RNase I_f_ treatment). R70 RTL (70 °C RTL with RNase I_f_ treatment) and 70RTL (70 °C RTL without RNase I_f_ treatment). The dsRNA was analyzed via R95 RTL, and R70 RTL and 70 RTL were assayed as negative control. The ssRNAs were analyzed by 70RTL, and R95 RTL and R70 RTL were used as negative control. Each standard point was analyzed via three technical replicates and the data was presented. The calculated dsRNA and ssRNA amount were back calculated via the equation of trend lines. The percent recovery is calculated from the measured RNA amount divided by the theoretical RNA amount

**Table 2:** RNase I_f_ -qPCR assay inter-assay precision

	dsRNA input for qPCR (pg)	Run 1 Cp	Run 2 Cp	Run 3 Cp	CV of CP	Average of CV
STD1	12.07	14.64	14.02	13.38	4.5%	5.0%
STD2	4.02	16.35	16.08	15.17	3.9%	
STD3	2.01	17.66	17.42	16.16	4.7%	
STD4	1.01	19.06	18.76	17.03	6.0%	
STD 5	0.50	20.52	20.13	18.67	4.9%	
STD6	0.25	21.82	21.68	19.95	4.9%	
STD7	0.13	22.59	22.07	19.79	6.9%	
STD8	0.06	24.57	24.55	23.03	3.7%	
STD9	0.03	24.71	24.77	22.26	6.0%	
STD 10	0.02	25.80	25.76	23.82	4.5%	

**Fig. 1:**
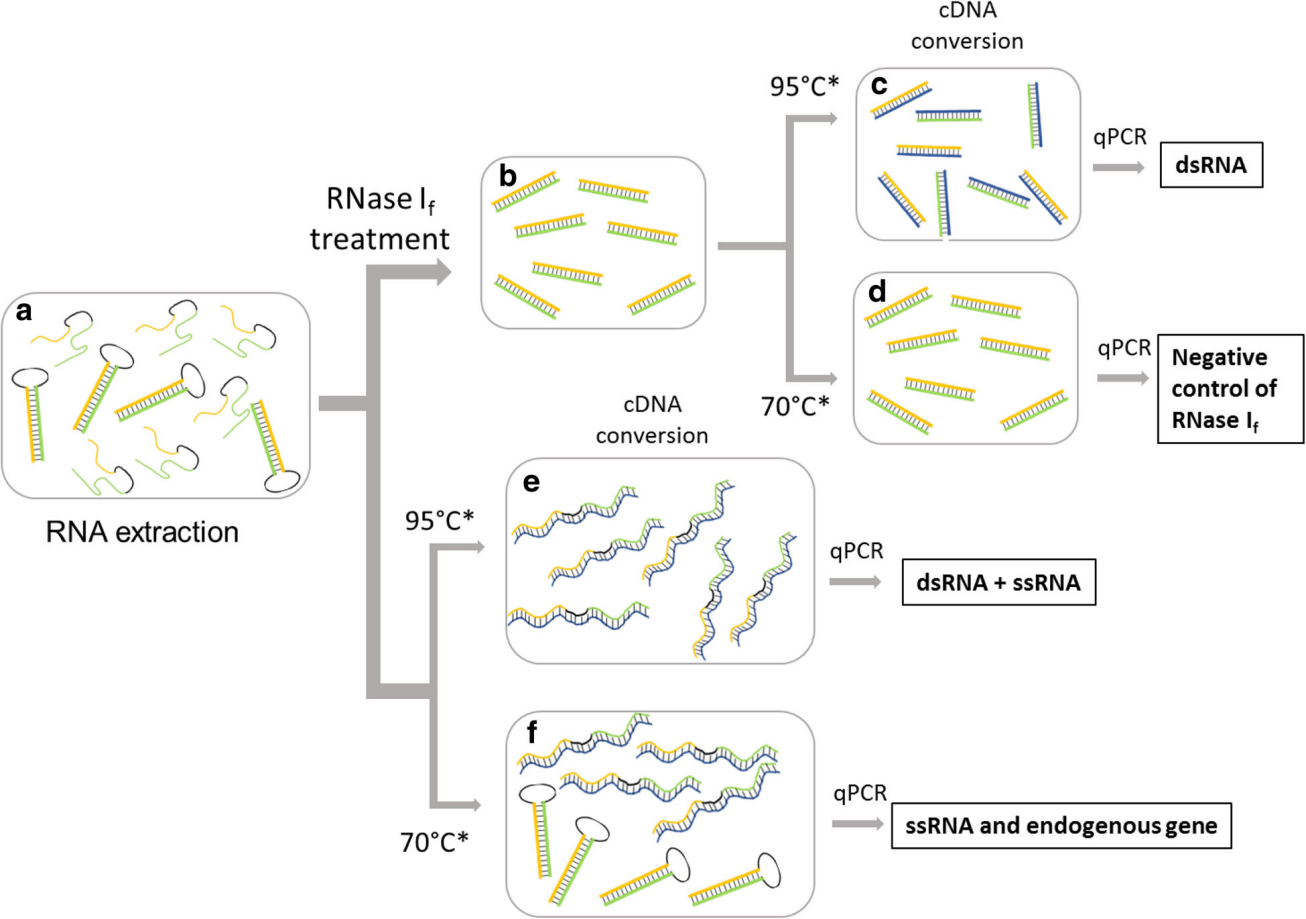
RNase I_f_ -qPCR assay overview. The total RNA is isolated first (**a**) using the protocol described in materials and methods. Then the isolated total RNAs are treated with RNase I_f_ and followed by RNA clean-up to obtain the purified dsRNA (loop sequence is digested) (**b**). The RNase I_f_ -treated RNAs are proceeded for cDNA conversion. The RNAs are pre-incubated with random hexamers at 95 °C (**c**) and 70 °C (**d**), respectively, and followed by cDNA conversion. *Blue* strands represent the reversely transcribed cDNA for the RNA incubated in 95 °C. In contrast, no cDNA is produced from the RNA incubated in 70 °C if all ssRNA is digested by RNase I_f_. In addition, non-RNase I_f_ -treated RNA was used for cDNA conversion at 95 °C (**e**) and 70 °C (**f**). For RNA pre-incubated in 95 °C. cDNAs (dark blue strand) are transcribed from both dsRNA and ssRNA. Oppositely, cDNAs are only converted from ssRNAs (including RNAi and endogenous gene). The qRT-PCR is performed after the cDNA conversion using the custom designed TaqMan™ assay

10-point standard synthetic v-ATPase C dsRNA was assayed in there independent runs by different analysts using the standard protocol described in Fig. 1. dsRNA amount (pg) in a 10 μL qPCR reaction and the corresponding mean (*N* = 3) Cp (crossing point-PCR-cycle) from each individual run are presented. The correlation between Cp and Log 2 RNA amounts are presented here. The coefficient of variation (CV) was calculated using the Cp values of each standard point

**Table 3:** RNase I_f_-qPCR assay to analyze distinct ratios of dsRNA and ssRNA mixtures

Mixture	dsRNA to ssRNA ratio	Means of dsRNA RTL	Means of ssRNA RTL	Expected dsRNA to ssRNA RTL ratio	Means of measured dsRNA to ssRNA RTL ratio^a^
1	1:5	31.27	148.52	0.20	0.21
2	2:7	25.49	75.07	0.29	0,34
3	3:8	21.44	45.67	0.38	0.47
4	1:2	21.92	38.30	0.50	0.57
5	1:1.5	17.67	23.29	0.67	0.76
6	10:1	567.75	56.41	10.00	10.06
7	5:1	115.43	23.21	5.00	4.97
8	7:2	85.94	22.33	3.50	3.85
9	1:1	48.00	37.15	1.00	1.29

RNase I_f_ -qPCR assay was performed to quantify the mixed synthetic dsRNA and ssRNA with distinct ratios indicated above, and the RTL of dsRNA and ssRNA was analyzed, respectively. Each mixtures were analyzed at least two technical replicates and the calculated mean ratios were presented. The dsRNA and ssRNA RTL were measured by R95 RTL and 70 RTL, respectively

^a^Chi-square analysis showed no significant difference between the expected and measured ratios

### Transgenic *Dv* v-ATPase C RNAi maize materials

The transgenic maize events producing *Dv* v-ATPase C hairpin RNA (hpRNA) were created based on the vector previously described [12]. Briefly, the binary vector carrying a 149-bp sequence (nucleotide positions 9-157 of *Dv* v-ATPase C open reading frame (ORF)) of *Dv* v-ATPase C sequence was constructed. The binary vector was designed to express a hpRNA with 149-bp invert-repeated sequence with a loop sequence in between the inverted repeats. Transcription of the v-ATPase C mRNA was driven by the maize *uniqutin* 1 promoter and terminated by a 3′ untranslated region (UTR) from a maize peroxidase 5 gene (ZmPer5 3’UTR). The binary vector was then transformed into *Agrobacterium tumefaciens*. The binary vector was transformed into maize via *Agrobacterium*-mediated transformation of immature embryos isolated from the inbred line, *Zea mays* c.v. B104 following the standard method [23].

### RNase I_f_ -qPCR

#### RNA extraction

Plant RNA extractions were carried out using the Norgen Plant/Fungi RNA purification kit (Norgen Biotek, Thorold, ON, Canada). Four leaf punches of maize plants at v4 stages were collected on ice into a 96-well collection plate. Samples were then ground in lysis buffer for 5 min using a 3.2 mm stainless steel bead. The supernatant was separated from the plant debris and precipitated with 100% ETOH. The subsequent binding and wash steps followed the manufacturer’s procedure with an on-column DNase I (Norgen) treatment for 15 min. Once eluted, the isolated RNA was then quantified on the NanoDrop-8000 (Thermo Fisher Scientific, Waltham, MA, USA) and normalized to 50 ng/μL.

#### RNase I_f t_reatment

The isolated RNA was digested with RNase I_f_ (New England Biolabs, Ipswich, MA, USA) per the manufacturer’s protocol at 37 °C for 10 min followed by heat inactivation at 70 °C for 20 min. The digested samples were purified using the RNA Clean-up and concentration kit (Norgen) following the manufacturer’s protocol. The RNase I_f_ treated and purified dsRNA were then used for cDNA synthesis.

#### First-strand cDNA synthesis

High capacity cDNA RT kit (Life Technologies, Carlsbad, CA, USA) was used for qRT-PCR analysis with the following modifications. (1) The dsRNA is pre-incubated at 95 °C and 70 °C for 5 min in the presence of the random hexamers and flash frozen with ice. (2) Multiscribe™ reverse transcriptase was then added to the pre-incubated RNA:hexamers mixture, then follow the manufacture protocol for cDNA conversion.

For the synthetic dsRNA/ssRNA mixture or leaf total RNA from transgenic plants, an aliquot of RNA was treated by RNase I_f_ to digest the ssRNA while another aliquot without RNase I_f_ treatment containing both the ssRNA and dsRNA was set aside. The same proportion of RNase I_f_ -treated and non-treated RNA was used for downstream cDNA conversion, respectively with the pre-incubation of RNA and random hexamers described above. For example, 1 μg of total RNA was digested via RNase I_f_ and eluted in 50 μL after clean-up. Five microliter (~ 100 ng) of RNaseIf-treated RNA was then used for cDNA conversion (both 95 °C and 70 °C); in a parallel set, 100 ng of untreated RNA was directly used for cDNA conversion (both 95 °C and 70 °C).

#### qRT-PCR

After the cDNA conversion using the RNA from different treatments, the ensuing cDNA was quantified via qRT-PCR using the LightCycler®480 system (Roche Diagnostics, Indianapolis, IN, USA) with a hydrolysis probe. The assays were run for the target gene (i.e., *Dv* v-ATPase C in this study) and the *Zea mays* TIP41 as the reference gene. For cDNA converted from the RNA without being treated by RNase I_f_, the assays were performed in biplex reaction (i.e., *Dv* v-ATPase C and TIP41). For cDNA from RNA treated with RNase I_f_, only *Dv* v-ATPase C was assayed in the singleplex reaction. To normalize the dsRNA, a separate reaction using the same proportion of RNA without RNase I_f_ treatment was used, as the TIP41 signal from a separate non RNase I_f_ - treated RNA was used to normalize dsRNA. To prepare the assays, LightCycler®480 Probes Master (Roche) was prepared at 1× final concentration in a 10 μL volume containing 0.4 μM of each primer and 0.2 μM of each probe. TIP41: (Forward primer: TGAGGGTAATGCCAACTGGTT; Reverse primer: GCAATGTAACCGAGTGT CTCTCAA and Probe: HEX-TTTTTGGCTTAGAGTTGATGGTGTACTGATGA-BHQ1). *Dv* v-ATPase C (Forward primer: GGGACACGATGAATAATATGACAAGTA; Reverse primer: CCAACTTTCAAGTCCGGGATT and Probe: FAM-CAACCAACTACAAGTTTC-MGB).

A two-step qRT-PCR program was performed with 40 cycles of the denaturation step at 95 °C for 10 s and extension step at 58 °C for 40 s with fluorescence acquisition. Cp values, the point at which the florescence signal crosses the background threshold using the fit points algorithm (LightCycler® software release 1.5) and the relative transcript level (RTL) was calculated using 2^ ^(−∆Ct)^ where ∆Ct = Ct (target gene) - Ct (reference gene) to obtain the relative quantitation over the endogenous gene.

### Northern blot

#### Sample preparation, digestion & denaturation

Synthetic ssRNA and dsRNA was diluted to 100 pg with 5 μg of wild-type maize B104 leaf total RNA. An aliquot of both was treated with RNase I_f_ to remove the ssRNA and another aliquot was left un-treated. The samples were then precipitated and resuspended in 20 μL of RNA sample loading buffer (Sigma-Aldrich, St. Louis, MO, USA). Five microliter (5 ng) of 0.3-1.5Kb DIG-labeled RNA molecular weight marker III (Roche) was aliquoted and added to 15 μL of RNA sample loading buffer. Samples and marker were denatured at 70 ͦ C for 15 min and immediately placed on ice.

#### Gel preparation and running

A 1.5% denaturing gel was created using Roche Mp agarose (Roche) with the addition of 6.5% formaldehyde in 1× MESA buffer (MOPS-EDTA-sodium acetate). Gel was cast and run in electrophoresis equipment capable of running samples at 65 V/30 mA for 2 h and 30 min.

#### Gel transfer

Following electrophoresis, the gel was rinsed in 2X SSC for 15 min. The gel was passively transferred to a nylon membrane (Millipore, Billerica, MA, USA) overnight at RT, using 10X SSC as the transfer buffer. Following the transfer, the membrane was rinsed in 2X SSC for 5 min, subsequently crosslinked (Stratagene, San Diego, CA), and allowed to dry at RT for up to 2 days.

#### Membrane hybridization, processing, and size determination

The membrane was prehybridized in UltraHyb buffer (Ambion) for 1-2 h at 65 °C. Blots described were probed with a 149-nt antisense single-stranded RNA complementary to the sense-strand RNA of the v-ATPase C hpRNA labelled with DIG digoxigenin-11-UTP using the Roche DIG RNA labeling Kit (Roche) according to manufacturer’s protocols [12]. Probes at a concentration of 80 ng/ml of hybridization solution were denatured at 95 °C for 5 min and placed on ice for at least 2 min before adding to fresh hybridization solution. Hybridization occurred overnight at a temperature of 65 °C in hybridization tubes. Following hybridization, the blots were subjected to the DIG immunological washes and detection (DIG wash and block buffer set, Roche) following manufacturer’s instructions. The wrapped blot was put up film for 1-20 min, developed and scanned.

### QuantiGene Plex 2.0 assay

The QuantiGene Plex 2.0 assay (QGP) assay (Affymetrix, Thermo Fisher Scientific, Waltham, MA, USA) used in this study was the custom triplex panel QGP Corn 41,669 3 Plx carrying three plex assay of *Dv* v-ATPase C, Elf1α and TIP like-41 (TIP41). One hundred twenty-five nanogram of total RNA from the same RNA source that was used for RNase I_f_ -qPCR was analyzed as assay input for QGP assay. The QGP assay was performed following the standard protocol of QGP except with the additional step to incubate RNA with probe sets. Briefly, the purified RNA was incubated with the probe sets at 95 °C for 5 min prior to mixing with the capture beads for overnight hybridization [21]. A typical probe set for a single target mRNA consists of a family of four or more different capture extenders (CEs), label extenders (LEs) and blocking probes (BPs). After the overnight hybridization, the analytes were washed of excess probes and remaining purified RNA, and the signal amplification reagents, consisting of the preamplifier (PreAmp), amplifiers (Amp), and label probes (LPs), were sequentially hybridized to the captured target RNAs via specific Luminex beads. The LP also includes a biotin molecule, which in turn is a binding site for the final signal amplification reagent, streptavidin-conjugated R-phycoerythrin (SAPE). Last, the Luminex 200 Flow Cytometer instrument was used to read the resulting fluorescence signal associated with individual capture beads.

The QGP signal is reported as median fluorescence intensity (MFI) and is proportional to the number of target RNA molecules extracted from the purified RNA of the samples. MFI was calculated by measuring signals on 35-100 beads/target. MFI values obtained from background blank wells with no target RNA were subtracted from MFI of each target reading. The relative quantification of *Dv* v-ATPase C was calculated by dividing the *Dv* v-ATPase C (MFI ATPase C - MFI background) to TIP41 (MFI TIP41 - MFI background).

## Results

### Method overview

The RNAi transgene design is composed of inverted-repeat sequences of the target gene inserted into the genome with a spacer loop region connecting the repeats. The mRNA transcripts from such transgenes are subject to form a self-complementary hairpin RNA (hpRNA) structure with a based-paired stem encoded by the inverted repeats and single stranded loop sequence. However, in addition to the hpRNA, the mRNA transcripts may sometimes form an unpredicted intermediate such as partial single-stranded mRNA (ssRNA). Because of the strong secondary structure of the hpRNA and the potential for unexpected ssRNA formation, challenges for reliable RNAi analytics during the development of RNAi trait could be encountered. Since the dsRNA strands are the active molecules for efficacy [11, 12], an analytical method which distinguishes dsRNA from ssRNA and effectively quantifies the dsRNA molecules is required for a RNAi trait development. To provide a reliable method to quantify the active RNA molecule (i.e., dsRNA longer than 60 bp) of an RNAi trait in transgenic plants, we developed the RNase I_f_ -qPCR assay. Figure 1 shows the assay overview scheme for the RNase I_f_ -qPCR assay (see protocol in methods). Briefly, RNA is first extracted from the plant samples. Then, the isolated RNA is aliquoted and treated with RNase I_f_ endonuclease to remove ssRNA, while an aliquot of RNA without RNase I_f_ was set aside. After the RNase I_f_ treatment, the remaining dsRNA (Fig. 1b) is further purified and converted to cDNA. For RNA aliquots treated with RNase I_f_, the RNA is pre-incubated with random hexamers at both 95 °C and 70 °C, and respective samples are converted to cDNA using standard protocols (Fig. 1c and d). The cDNA is then quantified using quantitative real-time PCR (qRT-PCR). The qRT-PCR result from the 95 °C pre-incubated cDNA conversion represents quantification of only dsRNA (abbreviation R95 RTL(relative transcript level)). The qRT-PCR result from the 70 °C pre-incubated cDNA conversion (abbreviation R70 RTL) serves as the negative control for RNase I_f_ treatment. If the RNase I_f_ treatment was effective, these samples (R70 RTL) should yield no detection as all the ssRNA should have been digested and the 70 °C is insufficient to open the dsRNA duplex for annealing of the random hexamers inside the dsRNA region being assayed. In addition to the RNase I_f_ -treated RNA, an equal amount of the non- RNase I_f_ -treated RNA is used directly for cDNA conversion with random hexamers at the same 95 °C and 70 °C pre-incubation. The result from the 70 °C pre-incubated cDNA conversion detects and quantifies the ssRNA present in the samples (abbreviation 70 RTL), which resembles regular qRT-PCR protocol. The 70 RTL and R95 RTL result may serve as a direct comparison between RNase I_f_ -qPCR and regular qPCR. The result from the 95 °C pre-incubated cDNA conversion represents the total of both the dsRNA and ssRNA present in the sample (abbreviation 95 RTL). Both ssRNA and dsRNA RTL were calculated using the endogenous gene quantified by 70 RTL treatment.

### RNase I_f_ -qPCR assay demonstrates dilutional linearity, accuracy and precision

**Fig. 2:**
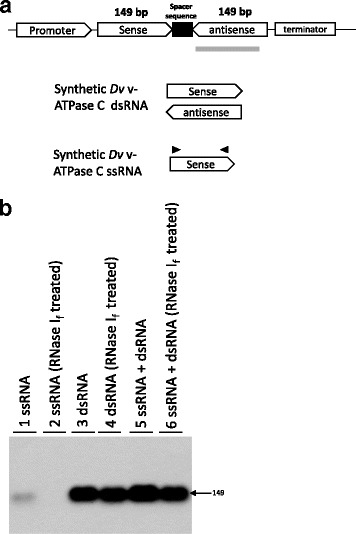
Northern blot to characterize the synthetic ssRNA and dsRNA. **a** RNAi targeted to *Dv* v-ATPase C transgene design cassette scheme. The transgene is comprised of a promoter and terminator between which an inversely-repeated sequence of the target gene is inserted with a spacer region between the repeats (promoter and terminator informaiton are described in Methods). Synthetic dsRNA and ssRNA are shown as diagram here. The thick *grey line* indicates the antisense RNA probe used for the RNA blot. The black arrows denote the primer location used in qRT-PCR. **b** Synthetic dsRNA and ssRNA (spiked into wild-type maize leaf B104 RNA) were treated with RNase I_f_. The non-treated RNA were used as control. The RNA blot was probed with the 149 bp DIG-labeled antisense RNA probe (shown in *gray bar* in A). The arrow on the right indicated the approximate molecular size

Since the whole procedure involves multiple steps, we first performed linearity testing to evaluate if the dsRNA or ssRNA analyzed by this method can display appropriate linearity. For this test, synthetic dsRNA and ssRNA were used for assay validation. A 149-bp sequence which targets the *Dv* v-ATPase C gene was selected for dsRNA synthesis. An ssRNA with overlapping region to the dsRNA was also synthesized (Fig. 2a). To confirm the secondary structure of dsRNA, a northern blot was used (Fig. 2b). The synthetic dsRNA and ssRNA were mixed with wild-type (WT) maize leaf RNA, separately or together, and then treated with RNase I_f_ to remove the ssRNA. RNA blot analysis for the ssRNA revealed no bands after the RNase I_f_ treatment, thus confirming complete ssRNA digestion via RNase I_f_ treatment. ssRNA was not detected after RNase I_f_ treatment, suggesting the capability of ssRNA digestion via RNase I_f_ (lane1 and 2). True to expectation, the dsRNA remained detected after the RNase I_f_ treatment with the RNA blot (lane3 and 4). This confirms the dsRNA duplex structure and validates the specificity of the RNase I_f_ treatment. The mixed dsRNA and ssRNA were still detected after the RNase I_f_ treatment, suggesting the dsRNA digestion is not affected in the presence of ssRNA (lane5 and 6).

**Fig. 3:**
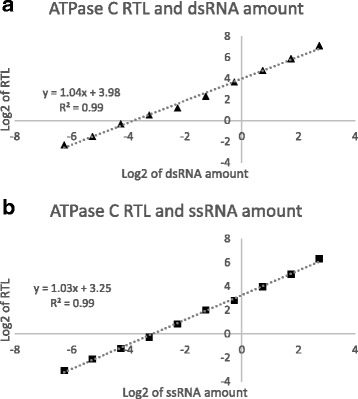
RNase I_f_ -qPCR assay linearity. 10-point standard of synthetic dsRNAs and sRNAs were analyzed using RNase I_f_ -qPCR described in Fig. 1. The synthetic RNAs were analyzed by 95 °C (RNase I_f_), and 70 °C (without RNase I_f_), to quantify dsRNAs (**a**), and ssRNAs (**b**), respectively. The relative transcript level (RTL) was obtained via normalization of target signal over the endogenous gene (TIP41). The correlation between log2 of RTL and RNA amounts are presented here. The equation of trend line and R^2^ is shown

To test for dilutional linearity, a 10-point dilution series of dsRNA and ssRNA were spiked into WT maize RNA, respectively. The samples were treated through the RNase I_f_ -qPCR assay (the corresponding dsRNA/ssRNA input in the final qRT-PCR is listed in Table 1). In the 10-point serial dilution of dsRNA, the R95 RTL to detect the dsRNA displayed dilutional linearity (Fig. 3a). The R70 RTL displayed no signal, indicating an effective RNase I_f_ digestion (Table 1). Much like for the dsRNA, the 10-point serial dilution of ssRNA, the 70 RTL to detect the ssRNA also displayed exceptional linearity (R^2^ 0.99) (Fig. 3b).

Using the 10-point dilution of synthetic dsRNA and ssRNA as outlined above, the post-extraction spike recovery was assessed, which may be inferred for the accuracy of the assay. The amount of 10-point dilution (~ 0.001 – 0.667 pg/ μL in the final qRT-PCR input), the corresponding RTL (R95 RTL, R70RTL and 70RTL) and % recovery are presented in Table 1. The accuracy of the assay is indicated as percent recovery that was calculated as a percentage of the back-calculated measurement against the standard curve over the theoretical amount. As shown in Table 1, the % recovery for each standard point is within the acceptance range (80 – 120%) except a few points slightly above (e.g. 121%) and below (75%). Altogether, the linearity testing suggests that the RNase I_f_ -qPCR assay demonstrates dilutional linearity with acceptable accuracy over a range of concentration. Also, the method demonstrates exceptional sensitivity. The limit of quantitation (LOQ) for *Dv* v-ATPase C dsRNA was determined from the lowest amount from the 10-point dilution as ~ 0.001 pg/ μL.

To further assess the repeatability of the assay (i.e., inter-assay precision), we created 10-point dilution of dsRNA and repeated the assays three times; each by different analysts. The Cp values from qRT-PCR of each standard point was captured and the coefficient of variation (% CV) was calculated as inter-assay precision using the Cp values of each standard point observed from three assay runs (Table 2 and Additional file 1: Figure S1). As shown in Table 2, average % CV among three independent runs are ~ 5% (acceptance criteria 20%), suggesting the acceptable repeatability for this assay. Based on the aforementioned tests in linearity, accuracy and precision, the results are in compliance with ICH guideline for validation analytical procedures [24].

### RNase I_f_ -qPCR assay determines the concordant ratios of dsRNA and ssRNA mixtures

The RNase I_f_ -qPCR assay is developed not only to effectively quantify dsRNA but also as a means to distinguish dsRNA from ssRNA. To test the assay for this capability, we mixed distinct ratios of dsRNA and ssRNA together with WT maize leaf RNA to resemble the varied amounts of dsRNA and ssRNA possible in transgenic plants and performed the RNase I_f_ -qPCR assay to test the accuracy of this assay. The dsRNA and ssRNA were measured from each distinct ratios of mixtures and the result are shown in Table 3. The nine different ratios of dsRNA and ssRNA mixtures, arranging from 1:5 to 10:1, were analyzed via RNase I_f_ -qPCR assay with at least two independent assays. As shown in Table 3, the RNase I_f_ -qPCR assay was able to determine the correct ratios of dsRNA and ssRNA for each mixture (Chi-square analysis showed no significant difference between the expected and measured ratios), demonstrating that the assay is capable of accurately measuring and distinguishing the dsRNA from ssRNA.

### The practical applicability of RNase I_f_ -qPCR assay to characterize transgenic maize materials

**Fig. 4:**
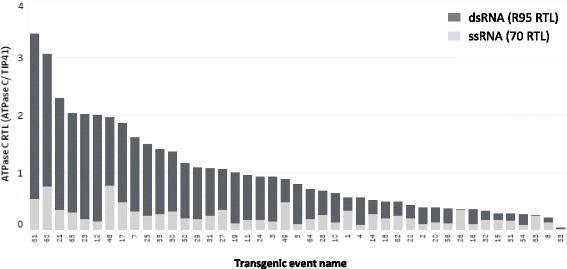
Characterization of *Dv* v-ATPase C RNAi expression in maize transgenic events using RNase I_f_ -qPCR. The total RNAs isolated from the leaf tissues of distinct transgenic maize T0 events carrying v-ATPase C RNAi. The RNAi transcripts were analyzed to quantify dsRNAs (R95 RTL) and ssRNAs (70 RTL), respectively. R70 RTL was assayed as control for RNase I_f_ treatment. The R70 RTL indicated effective RNase I_f_ treatment (data not shown). The RTL were normalized to endogenous gene (TIP41). The dsRNA and ssRNA RTL are represented as dark and light gray respectively

To demonstrate the practical applicability of this assay for deployment in trait development process, we tested transgenic maize containing an RNAi transgene. Transgenic events carrying *Dv* v-ATPase C RNAi were produced using the method described (details in materials and methods). RNA was isolated from leaf tissues of distinct T0 transgenic events, and were analyzed via the RNase I_f_ -qPCR assay to quantify the dsRNA and ssRNA RTL, respectively (Fig. 4). Within the distinct T0 transgenic events, different ratios of dsRNA and ssRNA were identified using this assay. As shown in Fig. 4, using this method, we could identify the varied expression levels of *Dv* v-ATPase C including both dsRNA and ssRNA: with some events having high abundance of dsRNA, while some exhibiting low expression, which is often observed in regular protein-coding trait a s well due to inserted genomic location. Importantly, using this method, we could distinguish the abundance of active molecules (i.e., dsRNA) from ssRNA, .

**Fig. 5:**
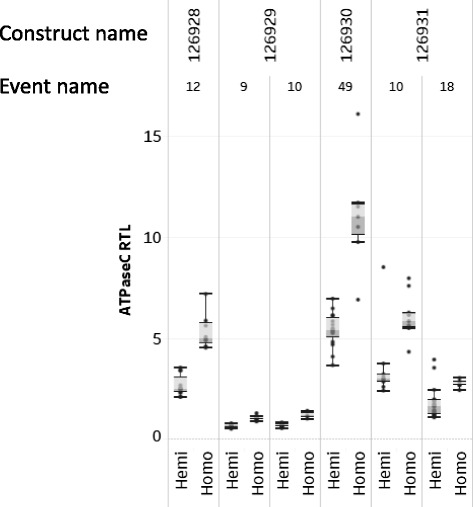
Investigation of dosage effect using RNase I_f_ -qPCR. The total RNAs isolated from distinct transgenic maize events from a segregating S1 population (including homozygous and hemizygous plants) of different constructs (listed on top) carrying v-ATPase C. The RNA were analyzed using the RNaseI_f_ - qPCR assay. The transcripts level were analyzed by 95 °C (RNase I_f_), and 70 °C (without RNase I_f_ treatment) to quantify v-ATPase C dsRNA, and the endogenous TIP41 gene, respectively. The v-ATPase C dsRNA RTL for each biological replicate was normalized to endogenous gene (TIP41). The mean RTL (*N* = 12) are denoted in the *box* plot

To further investigate the dosage effects using this method, we deployed the assay in a segregating S1 population including homozygous and hemizygous transgenic events. Distinct transgenic events from different constructs were assayed. In each transgenic event, the homozygous plants showed ~ 1.6-2.0 fold increase in dsRNA expression as compared to the corresponding hemizygous plants, suggesting that the assay may quantify the dsRNA with an accurate dose response (Fig. 5). Altogether, the assay as deployed on transgenic plants indicates that the RNase I_f_ -qPCR assay delivers reliable quantification to the active molecules of an RNAi transgene.

### Comparison of RNase I_f_ -qPCR assay quantification and QuantiGene Plex 2.0 RNA assay

**Fig. 6:**
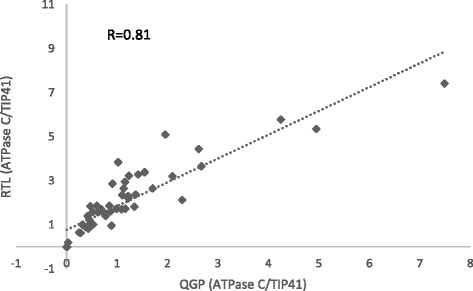
Cross comparison of RNase I_f_ -qPCR and QuantiGene Plex (QGP) assay. The purified leaf RNA samples from distinct transgenic maize T0 events are analyzed by RNase I_f_ -qPCR and QGP assay. The custom QGP assay detecting *Dv* v-ATPase C and TIP41 were performed and the data is shown on the x-axis. The RNase I_f_ -qPCR data is analyzed from the R95 RTL (RNase I_f_ treatment and cDNA from 95 °C incubation) and presented on the y-axis. The correlation coefficient R (*p* < 0.0001) is denoted

The utility of QuantiGene Plex 2.0 (QGP) platform for dsRNA detection has been previously shown [21]. The QuantiGene Plex 2.0 RNA assay is a platform that utilizes hybridization-based technology in which the target mRNAs are captured via custom designed probe sets coupled with magnetic microbeads. The detection of the signal results from the amplification of the captured oligonucleotides-RNA complex via branched DNA technology. To evaluate the concordance of QGP and the RNase I_f_ -qPCR, a custom QGP assay was designed based on the *Dv* v-ATPase C RNAi transgene in maize and the endogenous gene (e.g. TIP41). Purified leaf RNA from different transgenic maize events expressing *Dv* v-ATPase C RNAi transgene was used for cross comparison. RNase I_f_ -qPCR displays high concordance with QGP via the RTL measurements of the *Dv* v-ATPase C dsRNA expression among distinct transgenic events (Fig. 6). Regression analysis illustrates that RNase I_f_ -qPCR data had a significantly high correlation with QGP data: with the correlation coefficient R and slope for RNase I_f_ –qPCR versus QGP of 0.89 (*p* < 0.0001) and 1.07, respectively. Together, it suggests that the RNase I_f_ -qPCR assay can serve as an alternative analytical tool for dsRNA detection for RNAi trait in GM crops.

## Discussion

The application of RNAi in plants has been extensively explored in the past decades. Considering the application of RNAi for insect control was successfully demonstrated [3], this may lead to revolutionary benefits to agriculture for effective insect pest control with a distinct mode of action. Consequently, a reliable and precise analytical tool for RNAi is essential for RNAi trait development. In this study, we described a new modified qRT-PCR assay coupled with RNase I_f_ treatment. This new assay allows for the exclusive detection of the active RNAi molecules present in transgenes (i.e., dsRNA; > 60 bp). Through the assay validation process, we demonstrated that the assay displays dilutional linearity, precision and accuracy. Importantly, it can accurately distinguish between dsRNA from ssRNA generating concordant dsRNA-to-ssRNA ratios. This allows for the accurate quantitation of the active molecules (i.e., dsRNA) vs. non-efficacious intermediate (ssRNA). Applied to transgenic maize plants, the assay identifies events containing a significant level of *Dv* v-ATPase C RNAs which are present in single-stranded form (ssRNA). Without the ability to differentiate dsRNA from ssRNA, results may be misleading with inaccurate levels of active RNAi molecules (i.e., dsRNA). Thus, the described method here may serve as an effective tool to quantify dsRNA for RNAi traits in GM crops. In addition to the utilization of this method to characterize RNAi traits in crops, the reported method may be used for potential GM detection of the active ingredient as RNA-based approaches.

The success of this new dsRNA assay involves two critical steps: (1) Effective RNase I_f_ treatment and (2) 95 °C pre-incubation with random hexamers prior to cDNA conversion. Because it is important to ensure effective RNase I_f_ digestion, a QC step using 70 °C pre-incubation (R70 RTL) is important to evaluate if there are any undigested ssRNA remaining. Secondly, due to the strong intramolecular binding affinity of dsRNA, a 95 °C pre-incubation helps to ensure the dsRNA is open and accessible to the random hexamers prior to cDNA conversion. In addition to those two steps, because the RNase I_f_ -treated RNA lacks ssRNA, the RTL is normalized to the reference gene from a separate preparation produced without RNase I_f_ -treated RNA. Such cross normalization is not unprecedented used for qRT-PCR [25]. For traditional qRT-PCR using SYBR® Green I, cross normalization is required for relative quantification due to the singleplex nature of SYBR® Green I, and the results are still considered valid. In this assay, the equal proportions of RNA input for both the RNase I_f_ and non-RNase I_f_ cDNA conversions is particularly important in order to obtain reliable results due to the fact the RNA samples are treated separately. Overall, the method represents a cost-effective and feasible solution to quantify and differentiate dsRNA and may be leveraged into any application which requires for dsRNA quantitation.

## Conclusions

In conclusion, the RNase I_f_ -qPCR assay developed here provides an alternative method to effectively quantify the active molecules (i.e., dsRNA) of RNAi traits in GM crops. The modifications to conventional qPCR methods with the aid of a specific endonuclease and modified cDNA conversion protocol without additional equipment other than those used for conventional qPCR makes this method both feasible and cost-effective for all laboratories. This assay demonstrates equivalence to another hybridization-based method (i.e., QGP). Overall, the simplicity of this method makes it an alternative method for any application with the need of dsRNA quantitation such as the study of dsRNA virus in microbes.

## Additional file

Additional file 1: Figure S1. RNase I_f_ -qPCR assay inter-assay precision. A 10-point dilution of dsRNA was analyzed by different analysts for at least three times. Cp and log2 of dsRNA amount for each dilution were shown. (DOCX 42 kb)

## Abbreviations

dsRNA: Doubled-stranded RNA
GM: Genetic modified
hpRNA: Hairpin RNA
PCR: Polymerase chain reaction
QGP: QuantiGene Plex 2.0
RNAi: RNA interference
RNase I_f_ -qPCR: RNase I_f_ -treated quantitative real-time PCR
ssRNA: Single-stranded RNA
WCR: Western corn rootworm
