# Supplementary figures and images for: RNase If -treated quantitative PCR for dsRNA quantitation of RNAi trait in genetically modified crops
Source: BMC Biotechnol. 2018 Jan 17;18:3. doi: 10.1186/s12896-018-0413-6 (PMC5773123; doi:10.1186/s12896-018-0413-6)

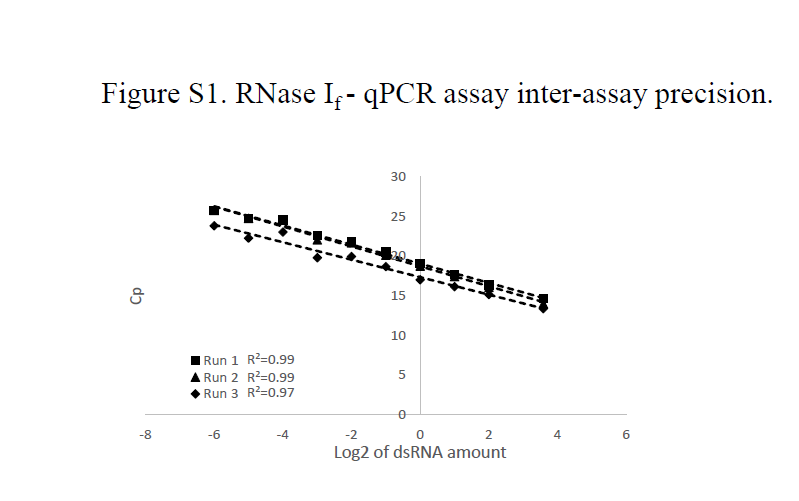

Supplement: Additional file 1: Figure S1. — RNase If -qPCR assay inter-assay precision. A 10-point dilution of dsRNA was analyzed by different analysts for at least three times. Cp and log2 of dsRNA amount for each dilution were shown. (DOCX 42 kb) [file 12896_2018_413_MOESM1_ESM.docx]
